# Predicting SSRI-Resistance: Clinical Features and tagSNPs Prediction Models Based on Support Vector Machine

**DOI:** 10.3389/fpsyt.2020.00493

**Published:** 2020-06-03

**Authors:** Huijie Zhang, Xianglu Li, Jianyue Pang, Xiaofeng Zhao, Suxia Cao, Xinyou Wang, Xingbang Wang, Hengfen Li

**Affiliations:** ^1^Department of Psychiatry, The First Affiliated Hospital of Zhengzhou University, Zhengzhou University, Zhengzhou, China; ^2^College of Economics and Management, Zhongyuan University of Technology, Zhengzhou, China; ^3^Department of Psychiatry, The Second Affiliated Hospital of Xinxiang Medical University, Xinxiang, China; ^4^Beijing Center for Health Development Studies, Beijing, China

**Keywords:** recurrent major depressive disorder, selective serotonin reuptake inhibitor-resistance, support vector machine, tagSNPs, prediction models

## Abstract

**Background:**

A large proportion of major depressive patients will experience recurring episodes. Many patients still do not response to available antidepressants. In order to meaningfully predict who will not respond to which antidepressant, it may be necessary to combine multiple biomarkers and clinical variables.

**Methods:**

Eight hundred fifty-seven patients with recurrent major depressive disorder who were followed up 3–10 years involved 32 variables including socio-demographic, clinical features, and SSRIs treatment features when they received the first treatment. Also, 34 tagSNPs related to 5-HT signaling pathway, were detected by using mass spectrometry analysis. The training samples which had 12 clinical variables and four tagSNPs with statistical differences were learned repeatedly to establish prediction models based on support vector machine (SVM).

**Results:**

Twelve clinical features (psychomotor retardation, psychotic symptoms, suicidality, weight loss, SSRIs average dose, first-course treatment response, sleep disturbance, residual symptoms, personality, onset age, frequency of episode, and duration) were found significantly difference (*P< 0.05*) between 302 SSRI-resistance and 304 SSRI non-resistance group. Ten SSRI-resistance predicting models were finally selected by using support vector machine, and our study found that mutations in tagSNPs increased the accuracy of these models to a certain degree.

**Conclusion:**

Using a data-driven machine learning method, we found 10 predictive models by mining existing clinical data, which might enable prospective identification of patients who are likely to resistance to SSRIs antidepressant.

## Introduction

Recurrent major depressive disorder (RMDD) is a clinical subtype of major depressive disorder (MDD) according to DSM-5 (1). Clinically, selective serotonin reuptake inhibitors (SSRIs) are commonly used in the treatment of MDD and the prevention of its recurrence (2). However, some studies have found that even if escitalopram plasma concentration reaches the therapeutic range, some individuals with MDD do not respond to the drug and the recurrence may not be prevented (3). Other studies have found that, no matter what kinds of SSRIs are taken, some patients never achieve satisfactory responses. I believe the phenomenon should be labelled as SSRI-resistance and therefore SSRI-R (4, 5). Notably, it takes more than two months and two treatment trials to determine whether a depressive patient is an SSRI-R (6). The time taken to identify SSRI-R has cost implications for clinical services, leading to the cost of drug therapy for depressive patients (7, 8). Therefore, being able to predict the outcome of SSRIs treatment at an early stage has significant benefits for treating MDD. According to the clinical symptoms of patients with MDD in clinical practices, some experienced psychiatrists try to subjectively predict if their patients with MDD will be SSRI-R, but that is not always successful and lacks objective criteria.

Recently, researchers have tried to utilize biological markers as objective predictors to predict the efficacy of SSRIs in the treatment of MDD. MDD is a complex disease (9).

Single nucleotide polymorphism (SNP), as a third-generation genetic marker, makes it possible to distinguish the characterizations of MDD between individuals and special community (10). SNPs are the most common type of human heritable variation; it accounts for more than 90% of all known polymorphisms (11). These genetic variations affect the individual’s differences in pharmacology by regulating gene expression (12). The SNPs with high information content which sufficiently represent haplotype diversity are called tagging SNPs, or simply referred to as tagSNPs (13). Since tagSNPs are representative and easily detected, selecting tagSNPs as research objects effectively reduces the research cost (14). The research interest on tagSNPs, which is a fixed SNP group label, arises. Studies about SSRIs resistance suggest that 5-HT-mediated adenylate cyclase (AC) - Cyclic adenosine monophosphate (cAMP) signaling pathway may play an important role in the mechanism of antidepressant therapy. Tsuchimine and Lisiecka, et al. report that depression may have abnormal regulation process mediated by cyclic adenosine monophosphate response element binding protein 1 (CREB1) and brain derived neurotrophic factor (BDNF) (15, 16). Although CREB1 and BDNF may be the targets of antidepressants, the findings from clinical studies are inconsistent (17–19). There are two main causes of this situation. First, clinical features possess complexity and lack reliable statistical analysis methods. Second, there is a lack of objective biological markers.

Based on the composition of complex data, advantages of machine learning are becoming increasingly prominent. Support Vector Machine (SVM) is a machine learning method based on statistical learning theory to apply to complex clinical data mining (20). It can apply clinicians’ valuable clinical experiences to clinic through information processing and data mining. SVM has unique advantages in small sample, non-linear, and high-dimensional pattern recognition (21). In present study, we sought to apply SVM to mine clinical features among 857 patients with RMDD who completed 3–10 years follow-up and their 34 tagSNPs involving 5-HT related signaling pathways to predict the SSRI-R at the early stage of MDD treatment.

## Methods

### Subjects

All participants with MDD were recruited from the inpatients or outpatients of the First Affiliated Hospital of Zhengzhou University and of the Second Affiliated Hospital of Xinxiang Medical University during the time from November 2005 to April 2014. The study was approved by the ethics committees from the two universities. All participants provided written informed consent. Inclusion criteria included: individuals with the diagnosis of recurrent major depressive episode according to DSM-IV, with score ≧21 of Hamilton Depression Rating Scale-24 items (HDRS-24), completing 3–10 year follow-up, aged 18–65 years, and male or female. All patients and their three generations were Chinese Han. Those who had comorbid psychotic illness, organic mental disorders, and psychoactive substance abuse were excluded. Of the 1,030 patients eligible from this study, 857 patients [83.2%, aged (39.17 ± 13.46) year old, 355 males and 502 females] received SSRIs and had more than three years follow-up data availability. The follow-up period ranged from 38 to 150 months, with a median of 50 months.

### Construction of Clinical Features Database

Clinical features data were obtained by the Structured Clinical Interview for DSM-IV Axis I, the items of HDRS-24 were applied for assessment of symptom severity, and the Eysenck Personality Questionnaire was assessed to personality. At more than 3-year follow-up (once every 2 to 4 weeks during depression episode, once every 2 to 3 months during remission), subjects were reassessed by means of diagnostic interviews from 2005 through 2014. There were three aspects related to database including socio-demographic, clinical features, and SSRIs treatment features during the first course treatment. Socio-demographic features included seven variables: gender (V1), age (V2), marital status (V3), education (V4), occupation (V5), personality (V6), and family history (V7). Clinical features included 16 variables: depressed mood (V8), loss of interest (V9), weight loss (V10), sleep disturbance (V11), psychomotor retardation (V12), fatigue (V13), negative thoughts (V14), loss of concentration (V15), suicidality (V16), circadian rhythm (V17), seasonal episodes (V18), sexual dysfunction (V19), psychotic symptoms (V20), age of onset (V21), frequency of episode (V22), and duration (V23). SSRIs treatment features during the first course treatment included nine variables: SSRIs average dose (V24), first-course treatment response (V25), sedation effect (V26), common adverse reaction (V27), rare adverse reaction (V28), residual symptom (V29), SSRIs non-response (V30), overdosage (V31), and combination of antidepressants (V32). (Definition and assignment of the variables are shown in the **Supplementary Table 1**). Among these variables, age of onset, frequency of episode, and duration are continuous variables, and the remaining are categorical variables.

### TagSNPs Gene Database

The SNPs were chosen according to the relevant literature. Applied with the NCBI database (HapMap Data Rel 28Phase II + III, August10, on NCBI B36 assembly, dbSNP bl26-CHB+JPT data), we carried out the preliminary tagSNPs selection (setting conditions: r2>0.8, MAF>0.001) and applied mass spectrometry analysis technique to complete the detection of 34 tagSNPs around 5-HT-mediated AC-cAMP pathway, and tagSNPs databases were constructed.

### SSRI-Resistance

Major depressive patients were given at least two SSRIs of adequate doses for 6 to 8 weeks (22, 23). The patients who did not respond to the treatment were defined as SSRI-R. The specific conditions were as follows: First, SSRIs dosage was no less than 40 mg/day for fluoxetine or its equivalent dose (sertraline ≧ 100 mg/day, paroxetine≧30 mg/day, fluvoxamine≧150 mg/day, citalopram≧15 mg/day, and escitalopram ≧ 15 mg/day). Second, adequate treatment was defined as having received SSRIs antidepressant agents for at least 6 weeks. Third, the reduction of HDRS-24 score was less than 25% compared to baseline (24). The patients who had more than 50% reduction of HDRS-24 score after 6-week treatment were called the SSRI non-resistance (SSRI-NR).

### General Data Processing

We analyzed data using the Statistical Package for the Social Sciences for Windows (version 21.0; SPSS). Variables are presented as either mean ± standard deviation (Mean ± SD) or frequency (%). We applied t-test or rank sum test and the chi-square (χ2) test to compare continuous and categorical variables between MDD patients with or without SSRIs resistance. Differences were considered statistically significant if *P < 0.05*.

### Data Mining and Analysis

We have used the libSVM learning software developed by Lin (25) (https://www.csie.ntu.edu.tw/∼cjlin/libsvm/). The process involved the following four steps. First, the type of kernel function to models was determined. The kernel function of SVM enables us to model higher dimensional, non-linear models. In a non-linear problem, a kernel function could be used to add additional dimensions to the raw data and thus make it a linear problem in the resulting higher dimensional space. Briefly, a kernel function could help do certain calculations faster which otherwise would need computations in high dimensional space. The polynomial kernel function was applied to establish the prediction model by SVM (by comparing the classification efficiency of four common kernel functions under the default parameters to find the optimal kernel function). Second, kernel parameter was optimized. The principle is that the training set is divided into K subsets. Each subset is regarded as a test set and the remaining subset sample is training set. That is, modeling K times, using the average absolute error of K times to evaluate the model performance. For each parameter pair (c, γ), cross validation was tried one by one, and then determined the optimal model parameters pair of the best accuracy rate of cross validation. Third, prediction model was established. Training samples were trained with SVM classifier with optimized parameters in order to obtain support vector, then determining SVM model. Lastly, we predicted test samples with the best model obtained by training. To eliminate the weight bias caused by the absolute value difference of data, the selected variables were normalized before the analysis.

## Results

### Clinical Variables Screening

**Figure 1:**
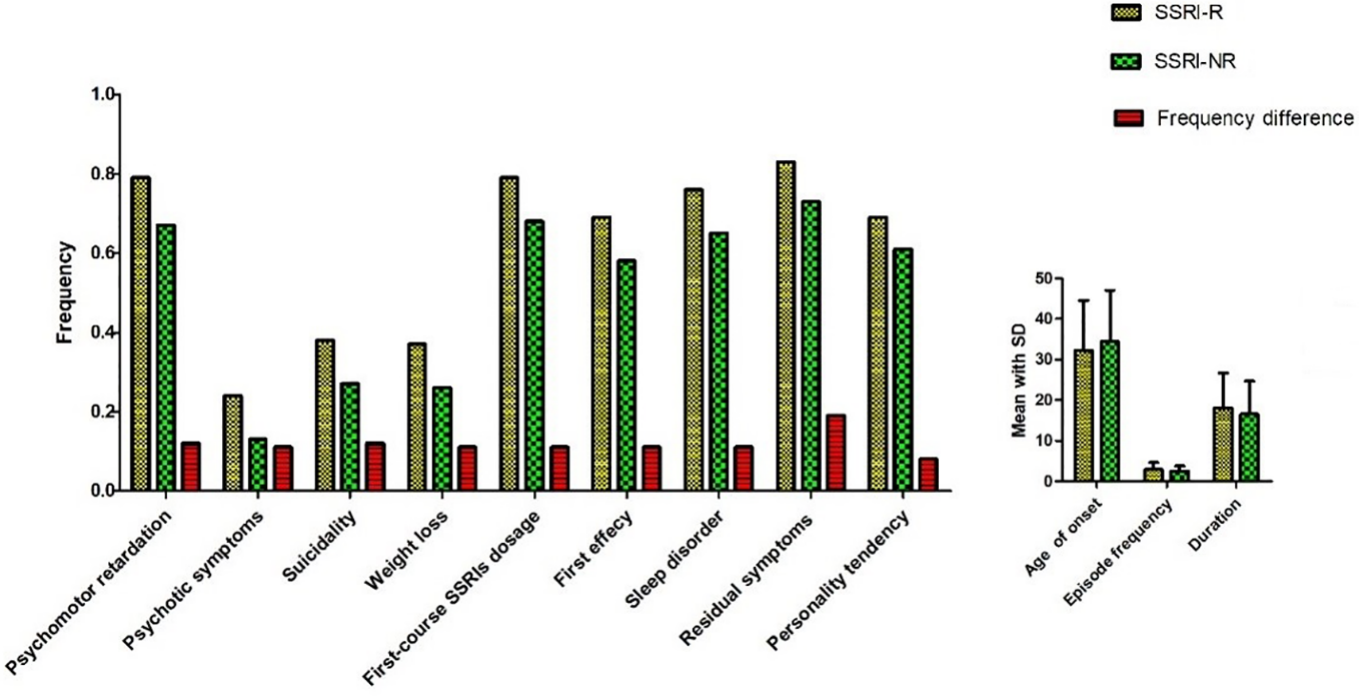
Comparison of clinical features between SSRI-R and SSRI-NR.

Eight hundred fifty-seven subjects were sequentially reordered by SSRIs treatment outcome (the reduction rate of HDRS-24 score) from low to high. Three hundred two patients (35.2%, 136 males and 166 females) were found to be SSRI-R. They were at the age of (39.5 ± 13.8) years old. HDRS-24 total scores were 21 to 66 (40.6 ± 8.8) before treatment. HDRS-24 total scores were 21 to 60 (34.2 ± 7.6) after treatment. Three hundred four patients (35.5%) met SSRI-NR. Among them, 121 were males and 183 were females, and their average age was (38.9 ± 13.1) years old. Total scores of HDRS-24 were 21 to 60 (40.0 ± 8.2) before treatment and 4 to 32 (10.2 ± 8.6) after treatment. By comparing clinical features, we found that there were significant differences in 12 clinical features between SSRI-R and SSRI-NR groups, including psychomotor retardation (χ2 = 11.068*, p=0.001*), psychotic symptoms (χ2 = 13.795*, p=0.000*), suicidality (χ2 = 9.559*, p=0.002*), weight loss (χ2 = 9.145*, p=0.002*), SSRIs average tolerance dose (χ2 = 10.049*, p=0.002*), first-course treatment response (χ2 = 25.343*, p=0.000)*, sleep disturbance (χ2 = 8.386*, p=0.004*), residual symptom (χ2 = 9.65*, p=0.002*), personality of Introverted neuroticism (χ2 = 18.091*, p=0.000*). In addition, there were statistically significant differences in three variables between the two groups, which were age of onset (*p=0.048*), frequency of episode (*p=0.031*), duration (*p=0.014*) (**Figure 1**, **Supplementary Table 2**).

### Parameters Optimization

Three hundred two SSRI-R patients and 304 SSRI-NR patients were mixed and divided into training samples and test samples in a ratio of 5:1. There were 505 training samples, including 254 SSRI-R patients and 251 SSRI-NR patients. There were 101 test samples, including 48 SSRI-R patients and 53 SSRI-NR patients. In this study, we applied multiple cross-validation and grid search to kernel parameters C and γ. The range of kernel parameters was in the region of log_2_ *C* =-3∼15, log_2_ *γ* =-15∼13. Accuracy of cross-validation was 59.60 to 90.38%.

### SSRI-R Predictive Model Screening

**Figure 2:**
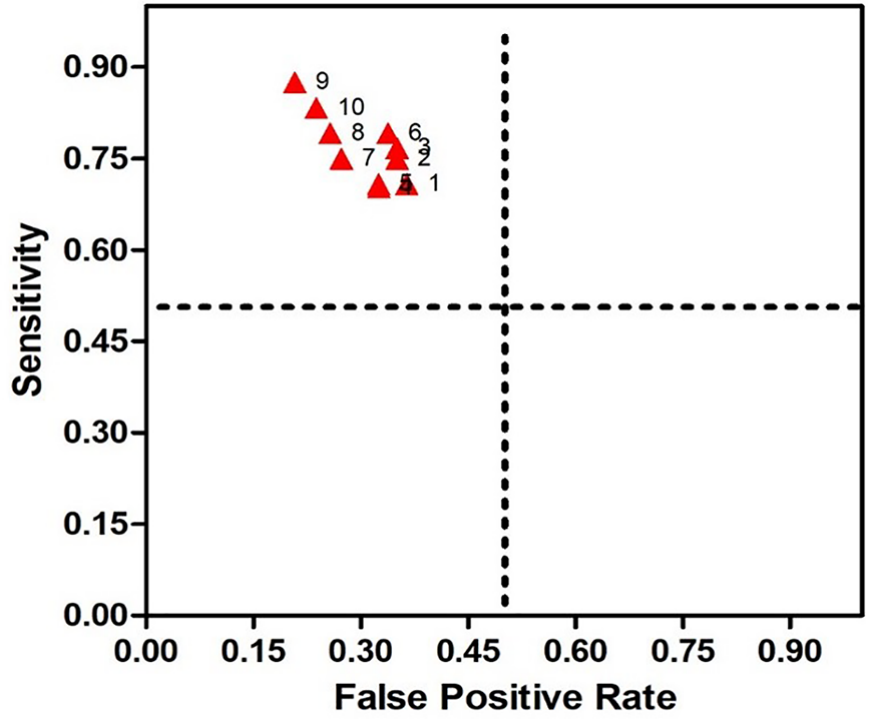
Receiver Operating Characteristic (ROC) for SSRI-R-PM. The sensitivity is illustrated on the y-axis, the false positive rate on the x-axis. Therefore, a dot above the diagonal line indicates better than random results, and the prediction results get better nearing the upper left comer.

The 12 clinical features mentioned above were regarded as primary predictive variables, and 11 queues $\left(C_{12}^{2}+C_{12}^{3}+C_{12}^{4}+C_{12}^{5}+C_{12}^{6}+C_{12}^{7}+C_{12}^{8}+C_{12}^{9}+C_{12}^{10}+C_{12}^{11}+C_{12}^{12}\right)$ were formed by random combination, including 4,083 combinations. Each combination in each queue was trained by SVM to get a prediction model, and models with high prediction accuracy (> 60.0%) for each queue were regarded as optimal prediction models of the queue. The results showed that prediction accuracy of 347 combinations ranged from 60.0 to 77.0%. In this study, we also measured other relevant descriptions of model discrimination-including sensitivity and specificity to evaluate the models. The combinations with sensitivity and specificity greater than 60.0% were selected as the optimal prediction models of this own queue. In addition to the $C_{12}^{2}$ queue, 10 prediction models were selected and named SSRI-R-PM 1 to 10, respectively. The accuracy, sensitivity, and specificity of SSRI-R-PM was 60.3–77.0%, 70.3–87.5%, and 63.6–79.2%, respectively (**Figure 2**). The kernel parameters and model variables are shown in **Table 1**.

**Table 1:** Model composition and parameters of SSRI-R Predictive Models.

SSRI-R-PM	Model composition	log_2_ *c*, γ	Accuracy (%)	Sensitivity (%)	Specificity (%)
SSRI-R-PM1	V20+V22	log_2_ *c* = 1 log_2_ *γ* = -1	(59/101) 58.4	70.8	60.4
SSRI-R-PM2	V20+V22+V23	log_2_ *c* = 1 log_2_ *γ* = -1	(61/101) 60.3	70.8	63.6
SSRI-R-PM3	V12+V16+V20+V23	log_2_ *c* = 3 log_2_ *γ* = -3	(63/101) 62.4	75.0	64.9
SSRI-R-PM4	V10+V12+V16+V20+V21	log_2_ *c* = 7 log_2_ *γ* = -11	(64/101) 63.4	76.7	649
SSRI-R-PM5	V10+V12+V16+V20+V21+V23	log_2_ *c* = 13 log_2_ *γ* = -5	(68/101) 67.3	70.3	67.5
SSRI-R-PM6	V10+V12+V16+V20+V21+V23+V24	log_2_ *c* = 5 log_2_ *γ* = -3	(71/101) 70.3	70.8	67.5
SSRI-R-PM7	V6+V10+V12+V16+V20+V21+V23+V24	log_2_ *c* = 7 log_2_ *γ* = -9	(75/101) 74.3	79.2	66.2
SSRI-R-PM8	V6+V11+V12+V16+V20+V21+V22+V23+V24	log_2_ *c* = 1 log_2_ *γ* = -3	(77/101) 76.2	75.0	72.7
SSRI-R-PM9	V6+V10+V11+V12+V16+V20+V21+V22+V23+V24	log_2_ *c* = 1 log_2_ *γ* = -3	(78/101) 77.2	79.2	74.3
SSRI-R-PM10	V6+V11+V12+V16+V20+V21+V22+V23+V24+V25+V28	log_2_ *c* = 1 log_2_ *γ* = -3	(77/101) 76.2	87.5	79.2
SSRI-R-PM11	V6+V10+V11+V12+V16+V20+V21+V22+V23+V24+V25+V28	log_2_ *c* = 1 log_2_ *γ* = -3	(77/101) 76.2	83.3	76.2

### The Influence of tagSNPs on SSRI-R Predictive Models

**Figure 3:**
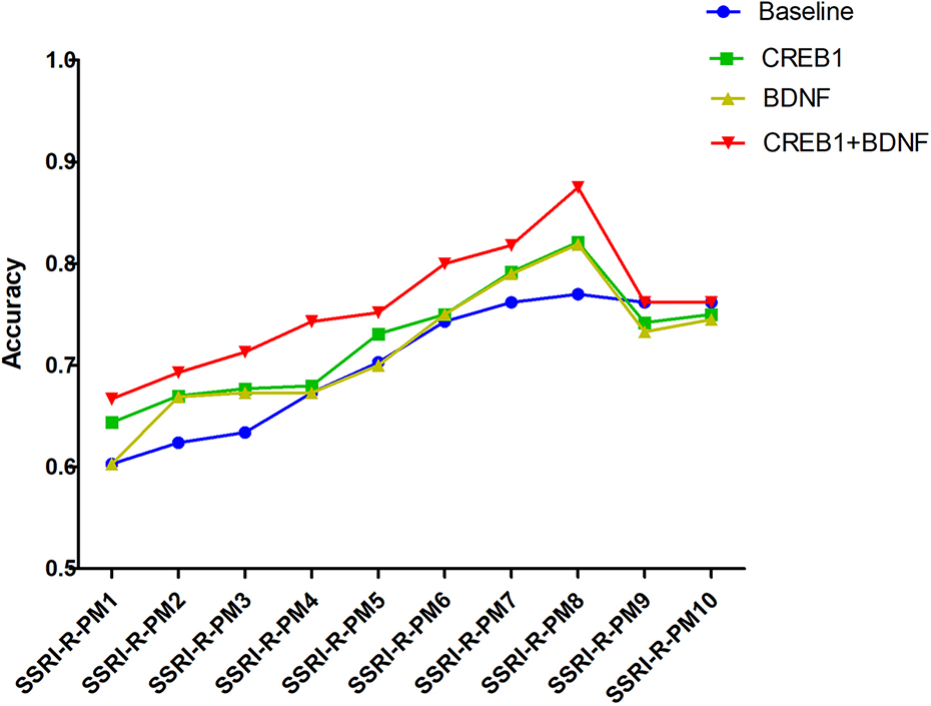
Accuracy of joint tagSNPs in SSRI-R-PM.

Compared to the frequency distribution of 34 tagSNPs genotype and alleles, the differences in the genotype distributions of four tagSNPs (CREB1: rs2551645 and rs4675690; BDNF: rs18035210 and rs7124442) between SSRI-R and SSRI-NR were statistically significant (*P<0.05*, **Supplementary Table 3**). Adding the two SNPs of CREB1, the two SNPs of BDNF, and the four SNPs of CREB1+BDNF into the 10 SRI-R-PM respectively, we found that adding CREB1+BDNF combined SNPs in SSRI-R-PM significantly increased the accuracy in SSRI-R-PM from 1 to 8, but not 9 and 10. The highest prediction accuracy (87.5%) was observed in SSRI-R-PM 8; 10.5% was increased from the model without the SNPs (**Figure 3**).

## Discussion

There may be different clinical features related to the SSRIs treatment outcome in different patients with RMDD. In this study, we found that 12 clinical features were significantly different between SSRI-R and SSRI-NR (*p < 0.05*), suggesting that those clinical features may be related to the SSRIs treatment outcome in the participants with RMDD. Our findings suggested that recurrent major depressive patients, who experienced young age of onset, higher number of depressive episodes, longer duration, and higher level of neuroticism and introversion, tended to be with SSRI-R. In addition, compared with SSRI-NR patients, SSRI-R patients with RMDD had higher proportion of psychomotor retardation, psychotic symptoms, and suicidality. Our findings were consistent with a European multicenter study on treatment resistant depression by Souery et al. (26). Our results also suggested that depression Subtypes maybe exist the heterogeneity in terms of RMDD (27, 28). Rantala et al. report that more refined subtype classification contributes to perfecting treatment options (29). Data from a 10-year study conducted by Kautzky indicate that non-response to the first antidepressant treatment increases the risk of treatment resistance and has good predictive function (30). The large Sequenced Treatment Alternatives to Relieve Depression (STAR*D) study shows that, while one-third of patients achieve remission from MDD after the initial treatment trail, more than 90% of these patients have at least 1 residual symptom (31). Patients with more residual symptoms after remission are more likely to relapse, which often shows poor responses to antidepressant treatment (30). Recent one study has found that insomnia was one of the most representative biobehavioral factors of greatest risk salience with depression (32). The inflammatory biotype induced by sleep disturbance may be a key phenomenon driving depression pathogenesis and recurrence, which often persists to serve as a potent predictor of depression recurrence (33). In this study, we also found that patients tolerated higher dosage of SSRIs in the first course of treatment might not better respond to SSRIs, but relevant studies were rare.

Generally, single variable was not able to predict the treatment outcome of SSRIs in RMDD. In concordance, increasing the number of factors was related to a higher accuracy in predicting the outcome of SSRIs treatment in RMDD, showing a cumulative effect of the predictors (34). A clinically significant prediction of outcomes could spare the frustration of trial and error approach and improve the outcomes of MDD through individualized treatment selection. In this study, we identified the demographic and clinical variables predicting the SSRIs treatment outcomes in 606 patients with RMDD. We developed predictive models in order to optimize the prediction of SSRIs treatment outcomes by SVM, and the interaction-based model of demographic and clinical variables significantly predicted SSRIs treatment outcomes.

Ten optimized predictive models were established to predict SSRIs treatment outcomes using SVM. The prediction accuracy, sensitivity, and specificity of these models were respectively 60.3–77.0%, 70.3–87.5%, 63.6–79.2%. Two of the ten models could provide theoretical evidences for early judgment about SSRIs treatment outcome. Predictive Model 2 to 5 with SSRI-R took early clinical features as the main predictors, such as psychomotor retardation, psychotic symptoms, suicidality, and weight loss. When these early clinical variables coexist, the accuracy of these models reached to about 70%, suggesting that the four models could be used for early judgment in advance with SSRI-R. Predictive Model 1 and 6 to 9 which brought into SSRIs treatment features during the first course treatment, repeated predictive variables with treatment resistant depression, such as higher recurrent tendency, average dosage, and longer duration. The contributing factors of treatment resistant depression were considerable complicated. We speculated that patients with treatment-resistant depression (TRD) could belong to SSRI-R high-risk individuals. We found that Predictive Model 9 added two more predictive variables than Model 7, namely treatment response to first antidepressant treatment and rare adverse reactions, the predictive accuracy almost remained unchanged, and we inferred that these two variables contributed less cumulative effect, even could not distinguish the contribution of single predictive variable. In 2014, Kudlow reported that antidepressants with different mechanisms might be a more effective conversion strategy for patients who had no response to SSRIs treatment firstly (35). However, a recent Mata analysis shows that the first sertraline treatment had no response completely (36). The “switch another antidepressant” strategy may be no better than simply leaving on sertraline. In this study, most of SSRI-R prediction models were mainly based on clinical data obtained from variables after medication or follow-up, so we inferred that partial features after first SSRIs treatment may be considered as evidences for evaluating SSRI-R.

TagSNPs added into SSRI-R predictive models could improve the accuracy of prediction. Four polymorphisms from CREB1 (rs2551645, rs4675690) and BDNF (rs10835210, rs7124442) genes were draw into SSRI-R predictive models above based on the previous researches. After the combination of the SNPs of CREB1 and BDNF, the results suggested that the accuracy of SSRI-R prediction models could be increased to some extent. CREB1 and BDNF combination mutations increased the risk of SSRI-R with RMDD patients, which maybe a potential biomarker for predicting SSRI-R. The accuracy of SSRI-R-PM8 increased to 87.5%, which suggested that clinical variables with Model 8 were more likely to SSRI-R if we combined CREB1 and BDNF mutation. Compared with GWAS (37, 38), SVM could better solve the related problems of polygenic recessive hereditary diseases by iterating data information of polygenic mutations based on SSRI-R predictive models. As a result, SSRI-R predictive models tagged by tagSNPs may provide more early and reliable practical evidences for screening SSRI-R individuals. However, our study also has some limitations. Some clinical data (e.g., frequency of episode, duration, and some SSRIs treatment features) were obtained by subjects’ self-reports and the retrospective assessment of these features could have led to recollection bias. Moreover, the restrictive exclusion criteria in the patient selection (e.g. psychoactive substance abuse) might have led to a well-defined study population that might be one of the neglected research samples. Meanwhile, we did not consider childhood trauma, inflammatory markers as well as neuroimaging features as possible SSRI-R predictors. Finally, during the process of finding adjustable factors which could really influence SSRIs treatment outcome, confounding factors may lead to instability estimates in machine learning (39, 40), and it was necessary to further modify the solution by expanding the sample quantity. In future research, these predictive models might be further enriched by adding neurobiological information such as neuroimaging-based or inflammatory markers (e.g. CRP) to continuously revise these SSRI-R prediction models. In conclusion, the early identification of MDD patients at high risk for SSRIs treatment resistance could guide clinicians in selecting optimal setting and intensity of care. Indeed, individuals at high SSRI-R risk could benefit from an early more aggressive treatment.

## Data Availability Statement

The raw data supporting the conclusions of this article will be made available by the authors, without undue reservation, to any qualified researcher.

## Ethics Statement

The studies involving human participants were reviewed and approved by Ethics Committee of the First Affiliated Hospital of Zhengzhou University. The patients/participants provided their written informed consent to participate in this study.

## Author Contributions

Conceived and designed the experiments: HZ, XL, and HL. Performed the experiments: JP, XZ, WC, XinyW, XingW. Analyzed the data: HZ and XL. Wrote the paper: HZ. All authors contributed to and have approved the final manuscript.

## Funding

This study was funded by the National Natural Science Foundation of China (81371494). The National Natural Science Foundation of China had no further role in study design; in the collection, analysis and interpretation of data; in the writing of the manuscript; and in the decision to submit the paper for publication.

## Conflict of Interest

The authors declare that the research was conducted in the absence of any commercial or financial relationships that could be construed as a potential conflict of interest.
