# Supplementary material for: Predicting SSRI-Resistance: Clinical Features and tagSNPs Prediction Models Based on Support Vector Machine
Source: Front Psychiatry. 2020 Jun 3;11:493. doi: 10.3389/fpsyt.2020.00493 (PMC7283444; doi:10.3389/fpsyt.2020.00493)
Supplement: Supplementary file 1 [file Table_1.pdf]

Supplementary Table 1. 32 variables variable definitions and assignments

Category	Variables	Definition	Assignment
Socio-demographic features	Gender（V1）		male=1 ，female=2
	Age（V2）		Year
	marital status (V3)		Unmarried=1, married =2
	Education (V4)	Whether or not high school education was the boundary	No=0，Yes=1
	Occupation (V5)	Whether patients were engaging in professional activities at present	No=0，Yes=1
	Personality (V6)	According to Eysenck personality scale (EPQ)	Extraversion=0，Introversion =1
	Family history (V7)	Whether three generations of patients has a history of mental disorder	No=0，Yes=1
Clinical features	Depressed mood (V8)	Reference to DSM-IV criteria	No=0，Yes=1
	Loss of interest (V9)	Reference to DSM-IV criteria	No=0，Yes=1
	Weight loss (V10)	Reference to DSM-IV criteria	No=0，Yes=1
	Sleep disturbance (V11)	Reference to DSM-IV criteria	No=0，Yes=1
	Psychomotor retardation, (V12)	Reference to DSM-IV criteria	No=0，Yes=1
	Fatigue (V13)	Reference to DSM-IV criteria	No=0，Yes=1
	Negative thoughts (V14)	Reference to DSM-IV criteria	No=0，Yes=1
	Loss of concentration (V15)	Reference to DSM-IV criteria	No=0，Yes=1
	Suicidality (V16)	Reference to DSM-IV criteria	No=0，Yes=1
	Circadian rhythm (V17)	Whether depressive symptoms have circadian rhythm changes	No=0，Yes=1
	seasonal episodes (V18)	Whether depressive episodes have seasonal patterns	No=0，Yes=1
	Sexual dysfunction (V19)	Whether patients lose their sex drive when depressed	No=0，Yes=1
	Psychotic symptoms (V20)	Reference to DSM-IV diagnostic criteria	No=0，Yes=1
	Age of onset (V21)	The age of the first depressive episode	Year

	Frequency of episode (V22)	Each episode conforms to the criteria of major depressive episode	Frequency
	Duration (V23)	The duration of each depressive episode	Week
SSRIs treatment features during the first course treatment	SSRIs average dose(V24)	Whether SSRIs dosage was no less than 40mg/ day for fluoxetine or its equivalent dose(Sertraline ≧ 100mg/day, Paroxetine≧30mg/day, Citalopram ≧ 15mg/day, Escitalopram ≧ 15mg/day or Fluvoxamine≧150mg/day)	No=0，Yes=1
	First-course treatment response(V25)	The response of SSRIs antidepressant treatment for the first course	Response=0 ，Nonresponse=1
	Sedation effect(V26)	Sedation effects such as excessive sleep a week before taking SSRIs antidepressants	No=0，Yes=1
	Common adverse reaction (V27)	Several symptoms-such as drowsiness, sexual dysfuction, headache, dizziness, stomach upset, weight loss, anxiety, insomnia, tremors and nausea occurred during the first 2 weeks of treatment.	No=0，Yes=1
	Rare adverse reaction (V28)	Serious adverse reactions-agranulocytosis, convulsions, extrapyramidal symptoms	No=0，Yes=1
	Residual symptom(V29)	Refer to partial remission after antidepressant treatment but not to clinical recovery. The Hamilton Depression Rating Scale (HDRS-24 items) ≧ 8.	No=0，Yes=1
	SSRIs nonresponse (V30)	According to the reduction of HDRS-24 score was less than 25% compared to baseline	No=0，Yes=1
	Overdosage (V31)	Whether the maximum antidepressant dosage of treatment was exceeded	No=0，Yes=1
	Combination antidepressants (V32)	At least two antidepressants were simultaneously taken.	No=0，Yes=1
