# Supplementary material for: Predicting SSRI-Resistance: Clinical Features and tagSNPs Prediction Models Based on Support Vector Machine
Source: Front Psychiatry. 2020 Jun 3;11:493. doi: 10.3389/fpsyt.2020.00493 (PMC7283444; doi:10.3389/fpsyt.2020.00493)
Supplement: Supplementary file 2 [file Table_2.pdf]

Supplementary Table 2.Clinical features of a sample of 606 patients treated for major depressive disorder according to treatment response after more than 6 weeks.

Clinical variables	SSRIs-R (n=302)	SSRIs-NR (n=304)	$\chi 2/t$	P value
Gender, Female, %	166（0.55）	183（0.61）	1.687	0.193
Age, years: mean±sd	39.50±13.79	38.85±13.14	0.609	0.605
Marital status, %	220（0.73）	231（0.76）	0.784	0.376
Educational level, %	189（0.63）	178（0.59）	1.030	0.310
Professional activity, %	185（0.61）	179（0.59）	0.357	0.550
Personality, Introversion, %	208（0.69）	158（0.61）	18.091	0.000
Family history, yes, %	69（0.23）	62（0.20）	0.538	0.463
Depressed mood yes, %	256（0.85）	248（0.82）	1.101	0.294
Loss of interest yes, %	250（0.83）	247（0.81）	0.241	0.624
Weight loss, yes, %	113（0.37）	79（0.26）	9.145	0.002
Sleep disturbance, yes, %	230（0.76）	199（0.65）	8.386	0.004
Psychomotor retardation, yes, %	238（0.79）	203（0.67）	11.068	0.001
Fatigue, yes, %	212（0.70）	221（0.75）	0.464	0.496
Negative thoughts, yes, %	267（0.88）	273（0.90）	0.302	0.582
Impaired attention, yes, %	203（0.67）	202（0.66）	0.041	0.840
Suicidality, yes, %	116（0.38）	81（027）	9.559	0.002
Circadian rhythm, yes, %	143（0.47）	133（0.44）	0.792	0.373
Seasonal episodes, yes, %	67（0.22）	50（0.16）	3.202	0.074
Sexual dysfunction, yes, %	115（0.38）	106（0.35）	0.674	0.412
Psychotic symptoms, yes, %	73（0.24）	38（0.13）	13.795	0.000
Age of onset, years, mean±sd	32.37±12.41	34.29±12.60	-2.114	0.048
Frequency of episode, mean±sd	2.90±1.73	2.51±1.24	3248	0.031
Duration, weeks, mean±sd	18.12±8.51	16.52±8.16	2.357	0.014
SSRIs average dose	239（0.79）	206（0.68）	10.049	0.002
First treatment response, yes, %	209（0.69）	177（0.58）	25.343	0.000
Sedation effect, yes, %	85（0.28）	91（0.30）	0.235	0.628

Common adverse reaction, yes, %	115（0.38）	101（0.33）	1.557	0.212
Rare adverse reaction, yes, %	9（0.03）	14（0.05）	1.096	0.295
Residual symptom, yes, %	252（0.83）	222（0.73）	9.650	0.002
Overdosage, yes, %	5（0.02）	—		
Combination antidepressants, %	69（0.23）	—		
