# Supplementary material for: Predicting SSRI-Resistance: Clinical Features and tagSNPs Prediction Models Based on Support Vector Machine
Source: Front Psychiatry. 2020 Jun 3;11:493. doi: 10.3389/fpsyt.2020.00493 (PMC7283444; doi:10.3389/fpsyt.2020.00493)
Supplement: Supplementary file 3 [file Table_3.pdf]

Supplementary Table 3. Frequency of genotypes and alleles in the SSRIs-R and SSRIs-NR groups.

tagSNP	group	genetype			Allele	
(gene)		χ^2 Values	p Values		χ^2 Values	p Values
		11	12	22	1	2
rs1064448	R(n=294)	21(0.07)	119(0.041)	154(0.52)	161(0.27)	427(0.73)
(ADCY7)	NR(n=300)	16(0.05)	122(0.41)	162(0.54)	154(0.26)	446(0.74)
		χ^2=0.855	*p=0.652*		χ^2=0.448	*p=0.503*
rs11676272	R（n=296)	60(0.20)	124(0.42)	112(0.38)	244(0.40)	360(0.60)
（ADCY3）	NR（n=299）	65(0.22)	143(0.48)	91(0.30)	273(0.46)	325(0.54)
		χ^2=3.709	*p=0.156*		χ^2=3.385	*p=0.066*
rs1967309	R(n=269)	40(0.15)	149(0.55)	80(0.30)	229(0.43)	309(0.57)
(ADCY9）	NR（n=273）	44(0.16)	147(0.54)	82(0.30)	235(0.43)	311(0.57)
		χ^2=0.199	*p=0.905*		χ^2=0.025	*p=0.874*
rs2531982	R(n=295)	36(0.14)	135(0.46)	124(0.40)	207(0.35)	383(0.65)
(ADCY9）	NR（n=297）	34(0.11)	138(0.49)	125(0.40)	206(0.35)	388(0.65)
		χ^2=0.087	*p=0.957*		χ^2=0.021	*p=0.884*
rs2531995	R(n=290)	140(0.48)	118(0.41)	32(0.11)	398(0.69)	182(0.31)
(ADCY9）	NR（n=301）	152(0.50)	120(0.40)	29(0.10)	424(0.70)	178(0.30)
		χ^2=0.453	*p=0.797*		χ^2=0.458	*p=0.499*
rs7196832	R(n=301)	28(0.09)	149(0.50)	124(0.41)	205(0.34)	397(0.66)
(ADCY9）	NR（n=302）	24(0.08)	126(0.42)	152(0.50)	174(0.29)	430(0.71)
		χ^2=5.070	*p=0.079*		χ^2=3.849	*p=0.050*
rs8061182	R(n=288)	61(0.21)	147(0.51)	80(0.28)	269(0.47)	307(0.53)
(ADCY9）	NR（n=299）	71(0.24)	155(0.52)	73(0.24)	297(0.50)	301(0.50)
		χ^2=1.084	*p=0.582*		χ^2=1.032	*p=0.310*
rs10835210	R(n=299)	168(0.56)	105(0.35)	26(0.09)	441(0.74)	157(0.26)
(BDNF)	NR（n=301）	139(0.46)	134(0.45)	28(0.09)	412(0.68)	190(0.32)
		χ^2= 6.326	***p=0.042****		χ^2=4.111	***p=0.043****
rs7124442	R(n=297)	9(0.03)	61(0.21)	227(0.76)	79(0.13)	515(0.87)

(BDNF)	NR（n=303）	2(0.01) 46(0.15) 255(0.84)	50(0.08) 556(0.92)
		χ^2=8.125 ***p=0.017****	χ^2=7.970 ***p=0.005****
rs6265	R（n=297)	84(0.28) 127(0.43) 86(0.29)	295(0.50) 299(0.50)
(BDNF）	NR（n=304）	73(0.24) 139(0.46) 92(0.30)	285(0.47) 323(0.53)
		χ^2=1.433 *p=0.488*	χ^2=0.936 *p=0.333*
rs7103411	R（n=301)	73(0.24) 141(0.47) 87(0.29)	287(0.48) 315(0.52)
（BDNF）	NR（n=299）	60(0.20) 155(0.52) 84(0.28)	275(0.46) 323(0.54)
		χ^2=1.979 *p=0.372*	χ^2=0.343 *p=0.558*
rs1123425	R(n=295)	39(0.13) 139(0.47) 117(0.40)	217(0.37) 373(0.63)
（NOS1）	NR（n=301）	54(0.18) 126(0.42) 121(0.40)	234(0.39) 368(0.61)
		χ^2=3.064 *p=0.216*	χ^2=0.554 *p=0.457*
rs2682826	R(n=297)	166(0.56) 117(0.39) 14(0.08)	449(0.76) 145(0.24)
（NOS1）	NR（n=300）	157(0.52) 116(0.39) 27(0.09)	430(0.72) 170(0.28)
		χ^2=4.362 *p=0.113*	χ^2=2.365 *p=0.124*
rs3741476	R(n=298)	165(0.56) 108(0.36) 25(0.08)	438(0.73) 158(0.27)
（NOS1）	NR（n=303）	152(0.50) 126(0.42) 25(0.08)	430(0.71) 176(0.29)
		χ^2=1.876 *p=0.391*	χ^2=0.961 *p=0.327*
rs527590	R(n=296)	112(0.39) 146(0.45) 38(0.16)	370(0.63) 222(0.37)
（NOS1）	NR（n=302）	118(0.34) 135(0.49) 49(0.17)	371(0.61) 233(0.39)
		χ^2=1.918 *p=0.383*	χ^2=0.147 *p=0.702*
rs7959232	R(n=278)	80(0.29) 127(0.46) 71(0.25)	287(0.52) 269(0.48)
（NOS1）	NR（n=269）	70(0.26) 108(0.40) 91(0.34)	248(0.46) 290(0.54)
		χ^2=4.525 *p=0.104*	χ^2=3.337 *p=0.068*
rs1047735	R(n=299)	60(0.20) 158(0.53) 81(0.27)	278(0.46) 320(0.54)
(NOS1）	NR（n=298）	56(0.19) 157(0.53) 85(0.28)	269(0.45) 327(0.55)
		χ^2=0.236 *p=0.889*	χ^2=0.220 *p=0.639*
rs3741475	R(n=302)	26(0.09) 116(0.38) 160(0.53)	168(0.28) 436(0.72)
(NOS1）	NR（n=298）	18(0.06) 126(0.42) 154(0.52)	162(0.27) 434(0.73)
		χ^2=1.956 *p=0.376*	χ^2=0.060 *p=0.806*

rs3770704	R(n=294)	174(0.59) 105(0.36) 15(0.05)	453(0.77) 135(0.23)
（CREB1）	NR（n=302）	184(0.61) 104(0.34) 14(0.05)	472(0.78) 132(0.22)
		χ^2=0.211 *p=0.900*	χ^2=0.209 *p=0.647*
rs4675690	R(n=292)	68(0.23) 111(0.38) 113(0.39)	247(0.42) 337(0.58)
（CREB1）	NR（n=296）	63(0.21) 142(0.48) 91(0.31)	268(0.45) 324(0.55)
		χ^2=6.335 ***p=0.042****	χ^2=1.058 *p=0.304*
rs2551645	R(n=300)	44(0.14) 113(0.38) 143(0.48)	201(0.34) 399(0.66)
（CREB1）	NR（n=302）	43(0.14) 143(0.47) 116(0.39)	229(0.38) 375(0.62)
		χ^2=6.335 ***p=0.042****	χ^2=2.554 *p=0.110*
rs2254137	R(n=299)	120(0.40) 142(0.47) 37(0.12)	382(0.64) 216(0.36)
(CREB1）	NR（n=303）	104(0.34) 151(0.50) 48(0.16)	359(0.59) 247(0.41)
		χ^2=2.816 *p=0.245*	χ^2=2.736 *p=0.098*
rs11839883	R(n=292)	174(0.60) 54(0.18) 64(0.22)	402(0.69) 182(0.31)
(CREB1）	NR（n=290）	167(0.58) 71(0.24) 52(0.18)	405(0.70) 175(0.30)
		χ^2=3.690 *p=0.158*	χ^2=0.135 *p=0.714*
rs1051738	R(n=290)	39(0.13) 16(0.06) 235(0.81)	94(0.16) 486(0.84)
(PDE4A）	NR（n=294）	29(0.10) 26(0.09) 239(0.81)	84(0.14) 504(0.86)
		χ^2=3.858 *p=0.145*	χ^2=0.834 *p=0.361*
rs7256677	R(n=299)	49(0.16) 159(0.53) 91(0.31)	257(0.43) 341(0.57)
(PDE4A)	NR（n=303）	62(0.20) 146(0.48) 95(0.31)	270(0.45) 336(0.55)
		χ^2=2.136 *p=0.344*	χ^2=0.304 *p=0.581*
rs878567	R(n=297)	11(0.04) 90(0.30) 196(0.66)	112(0.19) 482(0.81)
（HTR1A）	NR（n=301）	15(0.05) 99(0.33) 187(0.62)	129(0.21) 473(0.79)
		χ^2=1.229 *p=0.541*	χ^2=1.231 *p=0.267*
rs1328683	R(n=290)	45(0.16) 123(0.42) 122(0.42)	213(0.37) 367(0.63)
（HTR2A）	NR（n=291）	42(0.15) 117(0.40) 132(0.45)	201(0.35) 381(0.65)
		χ^2=0.645 *p=0.724*	χ^2=0.606 *p=0.436*
rs17068986	R(n=294)	86(0.29) 141(0.48) 67(0.23)	313(0.53) 275(0.47)
（HTR2A）	NR（n=300）	91(0.30) 150(0.50) 59(0.20)	332(0.55) 268(0.45)

		χ^2=0.867 *p=0.648*	χ^2=0.529 *p=0.467*
rs3125	R(n=287)	9(0.03) 68(0.24) 210(0.73)	86(0.15) 488(0.85)
（HTR2A）	NR（n=299）	15(0.05) 83(0.28) 201(0.67)	113(0.19) 485(0.81)
		χ^2=2.943 *p=0.230*	χ^2=3.182 *p=0.074*
rs9534495	R(n=298)	72(0.24) 138(0.46) 88(0.30)	282(0.47) 314(0.53)
（HTR2A）	NR（n=301）	81(0.27) 148(0.49) 72(0.24)	310(0.51) 292(0.49)
		χ^2=2.464 *p=0.292*	χ^2=2.093 *p=0.148*

Note: R stands for SSRIs resistance group, NR stands for SSRIs non-resistance group; 11 stands for TT or GG, 12 stands for TC or AG, 22 stands for CC or AA; 1 stands for T or G, and 2 stands for C or A.In this study, 34 tagSNPs related to ADCY7, ADCY9, ADCY3, BDNF, NOS1, PDE4A, HTR2A, CREB1 and HTR1A were detected, of which rs2059336 genotype only was TT in SSRIs-R and SSRIs-NR, and rs143117860 genotype only was CC. Type, rs2551926 are GG or CC, rs889895 are TT , the remaining 30 tagSNP results are ideal.
